# Designing a New Molecular Probe: The Potential Role for Tilmanocept (Lymphoseek^®^) in the Assessment of Patients with Painful Hip and Knee Joint Prostheses

**DOI:** 10.2174/1874325001711010212

**Published:** 2017-03-22

**Authors:** O.O. Adesanya, C.E. Hutchinson

**Affiliations:** Radiology department UHCW, Clifford Bridge Road. Coventry CV2 2DX, United Kingdom

**Keywords:** Joint prosthesis, Septic loosening, Aseptic loosening, Galium-68 Tilmanocept, PET-CT, SPECT-CT

## Abstract

**Background:**

There is a long history of nuclear medicine developments in orthopaedics beginning in the early 20^th^ century. Technetium-99m (^99m^Tc) has a short half-life of six hours, emits 140 keV gamma rays and is the most widely used isotope, imaged with the Anger (gamma) camera. Gamma image quality and test sensitivity in painful prosthetic joints can be improved with single photon emission computed tomography (SPECT) and SPECT/CT. Positron Emission Tomography-Computed Tomography (PET-CT) with Sodium Fluoride (^18^F-NaF) and ^18^Fluorine-fluorodeoxyglucose (^18^F-FDG) PET have promising and limited roles respectively in the investigation of painful prosthetic joints. New SPECT/CT and PET-CT isotopes targeting activated macrophages with ^99m^Tc Tilmanocept (Lymphoseek^®^) and ^68^Gallium labelled Tilmanocept respectively show potential as agents to demonstrate wear particles ingested by macrophages and multinucleated giant cells. An imaging algorithm using SPECT and/or PET agents is proffered as a cost effective way of speedily and accurately arriving a diagnosis.

**Methods:**

Review of the historical role of nuclear medicine in orthopaedics and research into the potential role of new radiopharmaceutical agents was undertaken. Guidelines and algorithms for the imaging of complicated joint prosthesis are provided.

**Results:**

There is an established role for nuclear medicine in orthopaedics and particularly in the investigation of complicated joint prostheses. Imaging with Tilmanocept provides new opportunities to shorten the time to diagnose loosened and infected joint prostheses.

**Conclusion:**

There is a potential new role for Tilmanocept, which can be utilised with both PET-CT and SPECT-CT technologies. Tilmanocept is a relatively new radiopharmaceutical which has a potential role in the imaging assessment of painful joint prosthesis.

Nuclear medicine imaging requires molecular probes which are concentrated or altered by specific biological processes being investigated [1] as well as a means of monitoring these biological probes [1]. Some of the very first attempts in designing radionuclides involved demonstrating skeletal metabolic change by Chiewicz and Treadwell using Phosphorus-32 and Strontium-89 respectively in the early 20^th^ century [2].

Over time, *In vitro* alpha, beta as well as gamma emissions from radioisotopes in animal tissue samples have been demonstrated using autoradiography, Geiger counters and or Sodium Iodide (NaI) crystal detectors [2].

With the use of autoradiography in the musculoskeletal system, isotopes have been incorporated into both the organic and inorganic components of animal bony skeleton. Cerium as well as Gallium-72 [3] have been deposited in osteoid tissues and Sulfur-35 labelled Chondroitin Sulfate [2]. Radium D and Thorium B were used to study mineral formation in bone and teeth [4].

Strontium-85, with a half-life of 65 days and a gamma emission of 514 KeV was the first isotope used in humans in 1956 and between 1956 and 1961 was used to demonstrate increased bone turnover in fractures, tumours, osteomyelitis, Paget's disease and breast cancer bone metastases [2]. The images were acquired 2 to 5 days post-injection using external counting with probes [2]. The rectilinear scanner was introduced in the early 1950s to assess thyroid disease and was later applied to skeletal disease. Image resolution was poor and lumbar and pelvic images took over 2 hours to acquire [2]. Strontium-87m has a shorter half-life of 2.8 hours and emits 388 keV photons, which is better suited to scanning than strontium-85 but the high soft tissue activity at the early imaging times, necessitated by its short half-life, result in inferior images [2].

Fluorine-18 (^18^F), a cyclotron produced positron emitter with a half-life of 110 minutes [5], is rapidly taken up by bone and clears rapidly from the soft tissues, resulting in high bone: background ratio [6] and the whole body could be imaged much more quickly with the rectilinear scanner but it had the disadvantage of being more expensive and had a limited availability [2]. Subsequently, Technetium-99m (^99m^Tc) labelled compounds were employed. Sodium fluoride Positron Emission Tomography-Computed Tomography (NaF PET-CT) bone imaging is making a resurgence partly due to the rapid proliferation of clinical PET-CT imaging since 2001 [7-9]. Coincident with the introduction of ^99m^Tc rectilinear photo-scanning systems were replaced gamma-ray scintillation/gamma cameras which were developed by Hal Anger in the late 1950s [10].

^99m^Tc is more widely available, cheaper and with a gamma emission of 140 keV which is more ideal for gamma camera imaging. It also has a short half-life of six hours [2]. These features enable higher activity injections in patients with a low radiation dose, and hence enable swift whole-body imaging [2]. Since 1971, bone scans have been performed with polyphosphates and then diphosphonates labelled with ^99m^Tc [2]. ^99m^Tc labelled bone scans are currently the most frequently performed study in nuclear medicine departments in the UK and are likely to be the biggest contributor to collective radiation dose from diagnostic nuclear medicine studies [11].

Experiments and advancements in translate-rotate motion of scintillation detectors by David Kuhl and Roy Edwards in the 1960s at the University of Pennsylvania led to the development of computed tomographic imaging, and tomography reconstruction algorithms which were applied to produce radionuclide single photon emission computed tomography (SPECT) [2, 12]. SPECT images of the spine and joints have significantly superior sensitivity than planar imaging [2]. Further improvements in test accuracy have come from hybrid fused physiological/anatomical imaging with SPECT/CT [2]. SPECT/CT provides clarification of the aetiology of indeterminate bone lesions and more correctly classifies bone tumours than with SPECT alone as well as averting the need to perform supplementary imaging [2].

PET provides cross-sectional images with high-contrast resolution and the ability to quantify levels of radioisotope uptake throughout the body [2]. PET was initially described by Hal Anger in the late 1950s and is based on the coincident detection of a pair of annihilated photons that are produced when emitted positron collide with electrons [2]. Positively charged positrons are emitted from the nucleus and have the same mass as electrons [2]. The first fused PET-CT in-line system with a positron emission tomographic (PET) scanner and a multi–detector row helical computed tomographic (CT) scanner combined as one machine was introduced in University Hospital Zurich 2001 [13]. Co-registered PET-CT images showed a significant improvement in lesion classification when compared with PET images alone [13]. ^18^Fluorine-fluorodeoxyglucose (^18^F-FDG) PET is used to image tumours as well as inflammatory and infectious diseases [2].

^18^Fluorine sodium fluoride (^18^F-NaF) is therefore making a return as a bone-imaging agent more than 40 years after its initial introduction before being surpassed by ^99m^Tc labelled diphosphonates [2]. The rapid proliferation of clinical PET imaging systems, has revived interest in this agent, which has several advantages over the ^99m^Tc diphosphonates [14, 15]. ^18^F^-^NaF does not bind to protein, has a higher capillary permeability and is rapidly cleared from blood resulting in a higher target-to-background ratio of ^18^F NaF as well as a twice higher bone uptake [2].

Skeletal conditions that can be imaged accurately with ^99m^Tc diphosphonates and ^18^F- NaF PET include fractures, avascular necrosis, complex regional pain syndrome (CRPS) or reflex sympathetic dystrophy [2] as well as benign bone lesions such as fibrous dysplasia, Paget’s disease, osteoid osteoma in addition to bone metastases. It can be difficult to distinguish progressive disease in metastatic bone disease from response to treatment using ^99m^Tc diphosphonates because of the flare response in the immediate aftermath of chemotherapy or hormonal treatment and which may last up to 6 months [2, 16], with this response being more exaggerated on ^18^F NaF PET [16]. ^18^F FDG PET more closely reflects successful chemotherapy or hormonal treatment response in bone metastases and also demonstrates extra-osseous malignant disease [2, 16]. ^18^F FDG PET is more sensitive for bone marrow and lytic metastases than sclerotic deposits [2, 16]. On the other hand, ^18^F NaF PET and ^99m^Tc -diphosphonates are more sensitive for sclerotic lesions and lesions with secondary reactive cortical change [2, 16, 17]. Although ^18^F FDG PET cannot completely obviate the need for bone biopsies because it cannot consistently distinguish malignant and benign skeletal lesions [2]. ^18^F FDG PET is useful for monitoring response to chemotherapy and hormone therapy but a 2 week time delay is required to avoid false negative results [2].

^18^F NaF PET-CT has been shown to be more accurate for detecting bone metastases than planar as well as SPECT Tc^99m^ bone scintigraphy and ^18^F NaF PET in patients with high-risk prostate cancer [18].

The three-phase bone scan, which analyses bone vascularity, capillary permeability and osteoblastic bone turnover, is a sensitive radionuclide test for diagnosing osteomyelitis, but this remains a nonspecific study [19] especially in the presence of fractures and orthopaedic hardware which can make image interpretation even more difficult [2, 19]. Combined, or sequential nuclear medicine imaging was therefore introduced to improve test specificity [2]: combined bone/Gallium studies (accuracy of 65%-80%); combined bone/leukocyte studies; combined labelled leukocyte/bone marrow imaging (accuracy of 90%); as well as combined bone/sulphur colloid studies [2]. Sulphur colloid marrow imaging helps map alterations in marrow distribution produced by orthopaedic hardware which may have resulted in false positive labelled leukocyte studies [2]. Labelled peptides and antigranulocyte antibodies [2].

^99m^Tc-Fanolesomab (NeutroSpec^^®^^) is a murine M class immunoglobulin that binds to the CD-15 antigen expressed on human leukocytes with sensitivity comparable to that of *in-vitro* labelled white cell scans [2], but serious side effects including patient deaths have led to its withdrawal [2]. On the other hand, Sulesomab (LeukoScan^^®^^) a murine monoclonal (Fab') antibody fragment of the lgG class is safer and does not induce a human anti-mouse antibody (HAMA) response [2] following the production of endogenous antibodies. LeukoScan^®^ contains mouse proteins, but anaphylactic and other hypersensitivity reactions incidents are less common [20].

The Fab' fragment of the IG1 antibody is freely soluble and crosses permeable capillary membranes and then binds to cross-reactive antigen-90 (NCA-90) on circulating neutrophils at infection sites [2]. Initial result suggested similar or better results than labelled white cell studies, but more recent studies show less accurate or rather variable results in diagnosing musculoskeletal infection [2, 20].

FDG-PET is less expensive for investigating bone infection than the use of combination imaging with labelled leukocyte/bone marrow/bone scans [2] and also hybrid imaging is generally more sensitive than conventional imaging [21]. However, the presence of prostheses produces mixed results [2] and other metal-ware can result in a reduced test specificity due partly to false positive results associated with foreign body reactions in aseptically loosened devices [22]. Initial attempts at human neutrophil imaging in bone infection with ^18^F FDG PET were initially thought to be promising [23], but ^18^F FDG PET has since been shown to be no more sensitive than three-phase bone scintigraphy and less sensitive than conventional radiography in detecting periprosthetic joint infection [22].

^99m^Tc-labelled Ciprofloxacin (Infecton^^®^^) was first postulated by Solanki [24]. Its physiological distribution does not include the normal bone marrow and this allows for evaluation of the spine and proximal limbs unlike radiolabelled white cell and Gallium-67 scans which demonstrate background bone marrow uptake [25]. Although initial reports for the use of ^99m^Tc-labelled Ciprofloxacin in infection were promising [24]. More recent accounts of the use of ^99m^Tc-labelled Ciprofloxacin show a reduced specificity for detecting of bacterial infections. Also the radioisotope physically disappeared from sites of infection and inflammation with equal rapidity [26]. Therefore, ^99m^Tc-labelled Ciprofloxacin has no role of in diagnosing orthopaedic infections [26].

## THE DEVELOPMENT OF NEW MOMLECULAR IMAGING PROBES

Radiopharmaceuticals labelled with positron emitting isotopes provide more favourable physical characteristics in radionuclide imaging than single photon isotopes [27]. On the other hand, radionuclides for single photon labelled compounds do provide a wider variety than PET agents and single photon labelled molecules also offer an important complimentary array of new possibilities in the development of new agents used in the process of drug development [27]. Other advantages of PET agents over SPECT agents are the higher sensitivity and more accurate quantification features [27], but PET agents are disadvantaged by their shorter half-lives and they are also less abundant as well as more expensive [27]. Single photon emission radionuclides on the other hand are foreign to the human physiology and biochemistry [28]. Exploratory studies for investigational new drugs (IND) which are also known as phase 0 studies [29] improve our understanding of toxicity and efficacy thus reducing clinical trial time, costs as well as the high failure rate of new drugs that occur during the traditional phases I to IV clinical trial stage. Phase 0 studies follow on from preclinical pharmacology and toxicology tests and occur before the typical Phase I clinical trial. Phase 0 studies may involve restricted human exposure to non-therapeutic quantities of the IND [29].

Radionuclide imaging with Positron Emission Tomography (PET) and Single-Photon Emission Computed Tomography (SPECT) enables speedier proof-of-concept testing and allows non-invasive visualization, characterization and quantification of biochemical processes that occur at cellular and subcellular levels in both humans and animals whilst only using nanomolar to picomolar concentrations of the IND [27].

Radionuclide imaging thereforeplays a big role in target identification and validation. The drug target can be a membrane, nuclear receptor, ion channel, enzyme, hormone, DNA or RNA molecule, or even unidentified biological entity [27].

Radionuclide imaging does not play a significant role in lead finding and optimization. This stage usually relies on *in vitro* drug target analysis with biochemical and cellular assays, *e.g.*, for compound purity, integrity, solubility, lipophilicity, safety, dissociation constant, permeability and target affinity [27].

Radionuclide imaging is used in compound profiling in animal models at the preclinical stage. In this, the investigative drug itself is labelled with an imaging probe for *In vivo* imaging using animal models of disease to provide valuable information concerning drug absorption, distribution, metabolism, elimination and efficacy (ADME). The images provide pharmacodynamics and biodistribution properties of a candidate drug [27].

Safety evaluation can involve imaging with animal studies for toxicology and determining the proper dose to be tested in the clinical trials. Many pharmaceutical companies have established their own imaging laboratories to do carry out these processes [27].

Clinical evaluation with SPECT involves clinical studies in 4 sequential phases (I–IV) after authorization by regulatory agencies [27].

## REGULATORY BODIES

Worldwide, nuclear Medicine guidelines are developed by a number international, continental, national as well as local regulatory organisations [30] and the introduction of new isotopes or the institution of new usages of old isotopes would have to navigate these guidelines and regulations. In Europe, the European Association of Nuclear Medicine (EANM) coordinates the development of guidelines, but also recognises the need for national guidelines. Likewise, In the United Kingdom, there are national regulations on the administration of radioactive substances but differences in clinical practice and service delivery mean that guidelines do not readily apply across regional and national boundaries [31, 32].

Some examples of the various statutory nuclear medicine guidelines and regulatory bodies in England include: Medicines and Healthcare products Regulatory Agency (MHRA) which regulates the use of new and established medicines and devices; the Carriage of Dangerous Goods and Use of Transportable Pressure Equipment Regulations 2009 regulates the transportation of radioactive substances; the Administration of Radioactive Substances Advisory Committee (ARSAC); the Radioactive Substances Act 1993 (RSA93) in Scotland, or the Environmental Permitting Regulations 2010 (EPR2010) in England and Wales which oversees the management of radioactive wastes and is policed by the Environment Agency; the Medical and Dental Guidance Notes which covers Ionising Radiation Regulations (IRR99) and is policed by the Health and Safety Executive (HSE). Lastly, there is the Ionising Radiation (Medical Exposure) Regulations (IRMER) 2000 with inspections carried out by the Care Quality Commission (CQC).

## TILMANOCEPT (LYMPHOSEEK^®^)

The diagnosis of aseptic loosening or infection of joint prosthesis may occur pre-operatively, intra-operatively or post-operatively [33]. Periprosthetic tissue histological and microbiological features are complimentary, but histopathology samples are more reliable than microbiological findings in distinguishing non-infected from infected revision arthroplasty tissues [34] because organisms are rarely isolated from microbiological cultures of periprosthetic tissues [34] and almost 11% of cases of false negative microbiological tests occur in septic loosening [34]. Furthermore, false positive microbiological cultures may also occur due to the growth of contaminants in aseptic loosening [34]. On the other hand, histology results may be uninterpretable due to underlying inflammatory joint disease such as rheumatoid arthritis [35].

Septic loosening is more reliably diagnosed when acute inflammatory infiltrates are demonstrated using permanent preparations of periprosthetic tissues with the presence of one or more neutrophil polymorphs per high power field (× 400) on average after the examination of at least 10 high power fields [34, 36]. In addition, histological assessments may also be carried out intraoperatively on cryostat sections [34].

There are several explanations for the relatively poor microbiological yield. Owing to the low numbers of bacteria in periprosthetic tissue samples, only a small percentage of samples from infected prostheses would be culture positive and gram-stains are less reliable [35]. In addition, the presence of comorbidities, low-virulence organisms, chronic infections and infections with fistulae as well as antibiotics or steroids therapy can reduce microbiological yield [37].

Periprosthetic sampling sensitivity can be increased by using polymerase chain reactions for the detection of universal 16S rRNA bacterial genes but this method can also produce false-positive results from necrotic bacteria [38]. However, by combining universal polymerase chain reactions with subsequent bacterial sequencing test specificity can be improved [38].

During joint revisions, surgical sampling of the prosthetic interface membrane yields better specimens for more accurate histopathological diagnosis of prosthetic joint infections than from specimens obtained from the pseudocapsule [39]. In a histopathological study of the periprosthetic membranes performed in 370 patients, 4 types of periprosthetic membranes have been shown to exist [40]:

1. Type I - Wear particle induced type (detection of microscopic foreign body particles; macrophages and multinucleated giant cells occupy at least 20% of the area [40, 41].
2. Type II - Infectious type (granulation tissue with neutrophilic granulocytes, plasma cells and few, if any, wear particles [40].
3. Type III - Combined type (aspects of type I and type II occur simultaneously [40].
4. Type IV - Indeterminate type (neither criteria for type I nor type II are fulfilled [40].

In the study, the incidence of histopathological membrane types were: type I (54.3%), type II (19.7%), type III (5.4%) and type IV (15.4%), but 5.1% of cases examined were not assessable [8]. The results showed a high correlation between histopathological and microbiological results [8].

Periprosthetic interface membrane microscopic particles consist of intra-operative contaminants from surgical tools, supplemental fixation wires, as well as remnants from surface processing [41]. More commonly, periprosthetic interface membrane microscopic particles consist of particulate debris created from wear and metal corrosion [41].

There are three main types of wear - adhesion, abrasion and fatigue [41] with the most common particle produced by wear in the periprosthetic tissues being polyethylene [41], with polymethylmethacrylate (PMMA) another common wear produced particle [41].

Metal implant surface corrosion occurs more commonly in metal-on-metal modular interfaces [41], producing particles which are metal-salt ion precipitates produced in the surrounding aqueous environment [41]. Chromium phosphate particulate debris is produced by corrosion and is the most commonly observed particle in the periprosthetic tissue specimens retrieved at joint revision [41]. Particulate debris spreads around periprosthetic regions *via* joint fluid reaching levels of billion particles per gram of tissue [41]. In addition, Chromium phosphate corrosion particles have been found at sites remote from joints [41], as well as Cobalt-alloy, and Titanium-alloy particles being other common corrosion particles produced [41].

Wear particles are under 1µm in size, and as such, cannot be visualised with light microscopy because the wavelength of visible light ranges from 0.4 to 0.7µm [41]. Because of this techniques for isolation, separation and characterisation of wear particles involves the digestion of periprosthetic tissue with proteolytic enzymes and an acid or alkali [41] making histopathological diagnosis more difficult.

Periprosthetic wear particles comprise ultra-high molecular weight polyethylene, bone cement, metallic and ceramic debris [42]. The particulate debris is phagocytosed by recruited and activated monocytes/macrophages provoking chronic inflammation which results in the with resultant bone loss around the prosthesis [42]. The osteolysis following proliferation, differentiation and activation of macrophages is thought to involve M1 macrophages which promote an inflammatory response early on following debris formation and subsequently to a lesser degree M2 macrophages which promote an anti-inflammatory response to promote bone healing, debris scavenging and angiogenesis [42]. This view is simplistic and even though there is a differential expression of M1 and M2 macrophages in periprosthetic tissues with a higher ratio of M1/M2 macrophages in debris-laden inflamed periprosthetic and pseudomembrane tissues [42], both M1 and M2 macrophages have both pro- and anti-inflammatory properties [43].

The ideal radiopharmaceutical for joint prosthesis using should be a labelled molecule or simple ion such as ^99m^Tc or ^18^F labelled for SPECT or PET imaging respectively. These isotopes have relatively short half-lives that allow larger amounts of radioactivity to be administered without increasing the patients’ absorbed dose [44].

The ideal radiopharmaceutical should be standardized and should require minimal preparation or modification prior to administration [45]. It should be chemically stable and should preferably injected intravenously in solution form to avoid bone marrow imaging [44]. As with radiopharmaceuticals in general, it should not elicit a pharmaceutical response or produce any chemical toxicity [44].

Single photon imaging using Tilmanocept can be performed with ^99m^TC labelled Tilmanocept (Lymphoseek^®^), which is a radiopharmaceutical comprised of a dextran backbone (10-kilodalton) to which multiple units of mannose and DTPA (dietylene triamine pentaacetic acid) are attached [46]. The mannose backbone binds to the mannose binding receptor CD206 (cluster of differentiation) receptor with a sub-nanomolar affinity [47] while DTPA binds to ^99m^Tc Lymphoseek^^®^^ is licenced for intradermal, subcutaneous, subareolar and peritumoural injection routes for lymphatic mapping in the localization of sentinel lymph nodes draining a primary tumours in a variety of cancers. Tilmanocept travels easier in lymphatic capillaries than blood capillaries because lymphatic capillaries are more permeable and larger than blood capillaries, thus enabling easier entry of these relatively large protein particles [48]. Theoretically, Lymphoseek^^®^^ binding in high concentration will provide evidence of high numbers of macrophages [46, 49]. ^99m^Tc Tilmanocept is FDA approved for use in assessing the nodal spread of squamous cell carcinoma in the head and neck [50].

There are ongoing trials using intravenous ^99m^Tc Tilmanocept such as in the evaluation of the safety of escalating doses of ^99m^Tc Tilmanocept by intravenous injection and skeletal joint imaging in patients with rheumatoid arthritis [51]. Another clinical study sponsored by Navidea^^®^^ Biopharmaceuticals and hosted by Massachusetts General Hospital is currently evaluating arterial Inflammation in patients infected with the Human Immunodeficiency Virus (HIV) following intravenous injection of ^99m^Tc Tilmanocept [52]. In the future, we hope that after intravenous use of Tilmanocept has been endorsed for clinical use, it could play a significant role in the evaluation of patients with painful joint prosthesis as an intravenous macrophage-seeking agent [53].

Intravenous ^99m^Tc Tilmanocept has been injected in animals [54] and has been shown to localise in synovial macrophages in mice with arthritis [55]. Furthermore, human synovial macrophages have demonstrated Tilmanocept accumulation *In vitro* [55].

Tilmanocept coincidence imaging using PET may potentially be performed with ^68^Gallium (^68^Ga) labelled Tilmanocept [56, 57]. ^68^Ga Tilmanocept is produced by chelating radioactive ^68^Ga to the diethylenetriaminepentaacetic acid (DTPA) moiety of the Tilmanocept molecule. ^68^Ga is produced by ^68^Germanium (^68^Ge)/^68^Ga generators that last serve as a stable source of ^68^Ga for more than one year [58] and has a half-life of 68 minutes and decays by emitting positrons [59]. Experimental studies have combined Tilmanocept with more widely available and cheaper ^18^F to produce ^18^F Tilmanocept for PET imaging [60]. However, formation of the C-F bond is a harsh laborious water sensitive process. Silica-based and boron-based aqueous ^18^F capture allows direct preparation of ^18^F Tilmanocept [60].

^68^Ga Tilmanocept demonstrates a dose-dependent rather low level of bone marrow uptake with peak SUVmax of approximately 1 to 2. Bone marrow-to-skeletal muscle uptake ratio is approximately 7. Uptake levels in skeletal muscle is significantly more than liver and lung uptake [47, 61]. ^68^Ga Tilmanocept is excreted into urine and also binds to CD206 receptors in mesangial cells within renal glomeruli [62]. A new concept of tri-modal imaging has been created using ^68^Ga and ^99m^Tc dual-labelled Tilmanocept combined with a fluorescent near-infrared dye (IRDye800CW) for PET, SPECT and optical imaging respectively [63]. *In vivo* Tilmanocept can play an important role in the demonstration of CD206 binding intracellular pathogenic organisms such as including bacteria, fungi, viruses and parasites as well as foreign materials [64]. Lymphoseek^®^ may perform as well as histologic analyses which require an extended time following prosthetic extraction to get an answer.

## FOLATE IMAGING

The beta folate receptor (FR-β isoform) is overexpressed on activated macrophages which accumulate at sites of inflammation and infection. Folate receptor avid isotopes have been shown to accumulate at sites of inflammation and could act as markers for inflammatory processes such as rheumatoid arthritis [65]. In addition, to the beta folate receptor (FR-β isoform), there are 3 other separate types of receptor polypeptides located in cell membranes (α, γ and δ) which also bind and endocytose folates and folate conjugates. The alpha folate receptor (FR-α isoform) demonstrates limited expression in normal tissue, mainly kidneys, choroid plexus, lungs, and placenta [66], but is upregulated in ovarian, uterine, brain, lung, kidney, breast as well as colorectal tumours [65].

PET and SPECT folate imaging can be performed with ^18^F Fluoro-deoxy-glucose folate PET [66] and ^99m^Tc-EC20 SPECT [66, 67] imaging respectively.

## SUMMARY

This paper shows that there is a potential new role for Lymphoseek, which is a relatively new radiopharmaceutical and that it may be used in the imaging assessment of painful joint prosthesis. The authors also show that Lymphoseek can be utilised with both PET-CT and SPECT-CT technologies.

It is well recognised the nuclear medicine studies have high negative predictive values [68] and also that a combination of more than one nuclear medicine study would result in a much higher positive predictive value [68]. The paper also shows that multimodality imaging which provides complimentary functional and anatomical data would result in improved diagnosis of the state of the periprosthetic membrane [69]. Therefore, a sensible combination of nuclear medicine studies using SPECT-CT or PET-CT is likely to be highly accurate.

The authors have therefore developed an imaging algorithm (Figs. **1a**-**e**) to reduce false negative and false positive cases. As discussed, the initial imaging of painful joint prosthesis with 3-phase ^99m^Tc bone SPECT-CT scans or ^18^F NaF PET-CT is able to both detect mechanical complications on the CT component as well as cases of infection and aseptic loosening on the radionuclide component (Figs. **1a**-**e**). The periprosthetic membranes may be assessed further for the presence of activated macrophages with Tilmanocept or Folate receptor imaging using either SPECT or PET imaging in patients with no evidence of mechanical complications or infection on their initial imaging. This is especially true in patients with predominantly late-phase positive isotope bone scans or late phase positive ^18^F NaF PET [14, 15]. The presence of a high concentration of macrophages would suggest particulate-induced wear (Type-I) or the combined type (Type III).

The infectious type (Type II) of periprosthetic membranes which consist of granulation tissue with neutrophilic granulocytes, plasma cells and few, if any, wear particles are usually obvious on 3-phase ^99m^Tc bone scans SPECT-CT and ^18^F NaF PET-CT, but this may be assessed further with labelled white cells or immunoglobulin fragments, using planar imaging or SPECT if required. Comparative costs of different relevant imaging investigations and their doses is presented in Table **1**.

## Figures and Tables

**Fig. (1a):**
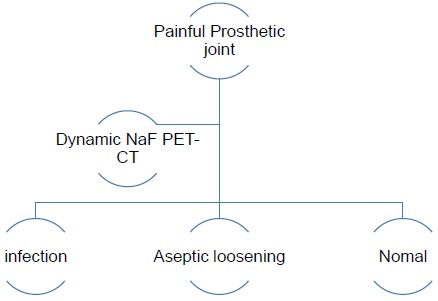
Dynamic PET-CT initial imaging of painful joint prosthesis – yielding results compatible with normal, aseptic loosening or infection.

**Fig. (1b):**
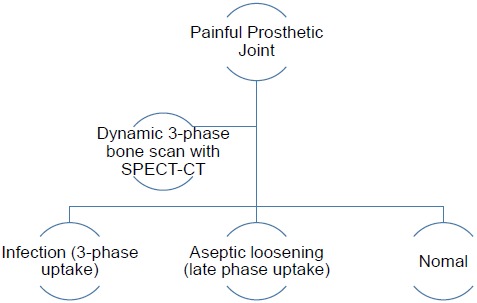
Dynamic SPECT-CT initial imaging of painful joint prosthesis – yielding results compatible with normal, aseptic loosening or infection (if no PET-CT is available).

**Fig. (1c):**
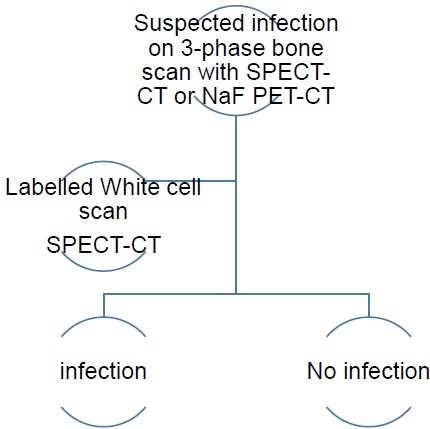
SPECT-CT labelled white cell imaging of suspected infected joint prosthesis – yielding results compatible with no infection or infection (if no PET-CT is available).

**Fig. (1d):**
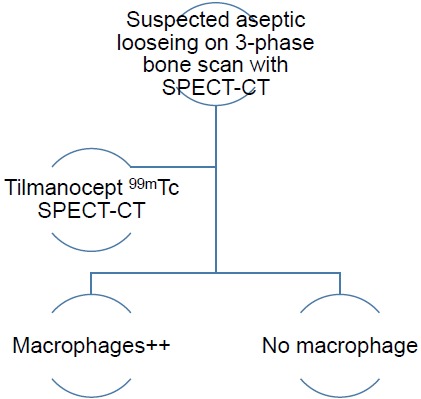
SPECT-CT Tilmanocept imaging of suspected aseptic loosening in joint prosthesis – yielding results compatible with a high macrophage burden or no macrophages (if no PET-CT is available).

**Fig. (1e):**
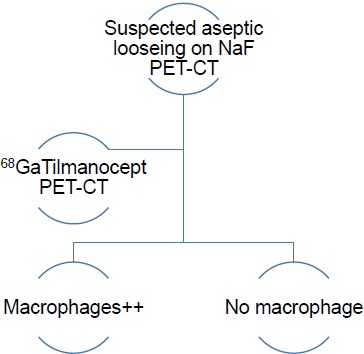
PET-CT Tilmanocept imaging of suspected aseptic loosening in joint prosthesis – yielding results compatible with a high macrophage burden or no macrophages.

**Table 1:** Typical cost and radiation dose of commonly performed examinations.

**Diagnostic procedure**	**Typical effective dose (mSv)**	**Equivalent number of chest X-rays**	**Approx equivalent period of natural background radiation**	**Typical Cost**
**Radiography (Plain Film)**				
Limbs and joints (except hip)	<0.01	<1	<2 days	**£**
Pelvis	0.3	20	1.5 months	**£**
**Ultrasound**				
Ultrasound joint	0	0	0	**£**
**Computed Tomography (CT)**				
CT pelvis	6	370	2.5 years	**£ £**
**Magnetic Resonance Imaging (MRI)**				
MRI limb	0	0	0	**£ £ £**
**Radionuclide (Nuclear Medicine)**				
Bone (Tc-99m-HDP)	3	200	1.3 years	£
PET-CT body (NaF or F-18 FDG)	18	1200	8.1 years	£ £ £ £

£ - <£35 ££ - £35-45 £££ - £45-60££££ - >£60 Some variances: 1 vial of Lymphoseek^®^ costs circa £1,175 (because this is a new patented radiopharmaceutical agent). A new PET-CT machine with service contract in place will cost approximately £1.6 million. A modern SPECT-CT gamma camera machine with diagnostic CT capability and service contract in place will cost approximately £ 0.5 million.
